# Comparison of Optimised MDI versus Pumps with or without Sensors in Severe Hypoglycaemia (the Hypo COMPaSS trial)

**DOI:** 10.1186/1472-6823-12-33

**Published:** 2012-12-13

**Authors:** Stuart Little, Thomas Chadwick, Pratik Choudhary, Cath Brennand, Julia Stickland, Shalleen Barendse, Tolulope Olateju, Lalantha Leelarathna, Emma Walkinshaw, Horng K Tan, Sally M Marshall, Reena M Thomas, Simon Heller, Mark Evans, David Kerr, Daniel Flanagan, Jane Speight, James AM Shaw

**Affiliations:** 1 Institute of Cellular Medicine, The Medical School, Newcastle University, 4th Floor William Leech Building, Framlington Place, Newcastle upon Tyne NE2 4HH, UK; 2 Institute of Health and Society, Newcastle University, Newcastle, UK; 3 Diabetes and Nutritional Sciences Division, Kings College London, London, UK; 4 AHP Research, Hornchurch, UK; 5 Bournemouth Diabetes Centre, Bournemouth, UK; 6 Institute of Metabolic Science, University of Cambridge, Cambridge, UK; 7 School of Medicine and Biomedical Sciences, Sheffield University, Sheffield, UK; 8 Peninsula College of Medicine and Dentistry, Plymouth, UK; 9 The Australian Centre for Behavioural Research in Diabetes, Diabetes Australia – Vic, Melbourne, Australia; 10 Centre for Mental Health and Wellbeing Research, School of Psychology, Deakin University, Burwood, Australia

## Abstract

**Background:**

Severe hypoglycaemia (SH) is one of the most feared complications of type 1 diabetes (T1DM) with a reported prevalence of nearly 40%. In randomized trials of Multiple Daily Injections (MDI) and Continuous Subcutaneous Insulin Infusion (CSII) therapy there is a possible benefit of CSII in reducing SH. However few trials have used basal insulin analogues as the basal insulin in the MDI group and individuals with established SH have often been excluded from prospective studies. In published studies investigating the effect of Real Time Continuous Glucose Monitoring (RT-CGM) benefit in terms of reduced SH has not yet been demonstrated. The primary objective of this study is to elucidate whether in people with T1DM complicated by impaired awareness of hypoglycaemia (IAH), rigorous prevention of biochemical hypoglycaemia using optimized existing self-management technology and educational support will restore awareness and reduce risk of recurrent SH.

**Methods/design:**

This is a multicentre prospective RCT comparing hypoglycaemia avoidance with optimized MDI and CSII with or without RT-CGM in a 2×2 factorial design in people with type 1 diabetes who have IAH. The primary outcome measure for this study is the difference in IAH (Gold score) at 24 weeks. Secondary outcomes include biomedical measures such as HbA1c, SH incidence, blinded CGM analysis, self monitored blood glucose (SMBG) and response to hypoglycaemia in gold standard clamp studies. Psychosocial measures including well-being and quality of life will also be assessed using several validated and novel measures. Analysis will be on an intention-to-treat basis.

**Discussion:**

Most existing RCTs using this study’s interventions have been powered for change in HbA1c rather than IAH or SH. This trial will demonstrate whether IAH can be reversed and SH prevented in people with T1DM in even those at highest risk by using optimized conventional management and existing technology.

**Trial Registration:**

ISRCTN52164803 Eudract No: 2009-015396-27

## Background

Type 1 diabetes mellitus (T1DM) accounts for 5–10% [1] of those with diabetes and is characterised by an absolute deficiency of insulin caused by immunologically mediated damage to the beta-cells in the pancreas. Onset can occur at any age but is most commonly in children, adolescents and young adults. Complications include microvascular disease of the kidneys, eyes and nervous tissue in addition to macrovasular disease such as ischaemic heart disease, cerebrovascular disease and peripheral vascular disease.

As there is no cure for T1DM, management entails regulation of blood glucose levels with insulin replacement therapy and dietary modification. There is incontrovertible evidence from the landmark DCCT [2] and the follow-up EDIC [3] study that microvascular and macrovascular complications can be prevented by rigorous avoidance of high glucose levels.

Despite 90 years’ clinical experience with insulin replacement therapy, however, severe hypoglycaemia (SH) remains the major factor limiting optimal glycaemic control [4]. In a retrospective epidemiological survey of an unselected population with T1DM the prevalence of SH was reported to be 37% over a one year recall period [5]. SH remains one of the most feared complications of insulin therapy as it can result in collapse without warning, fits, or even sudden death [6,7]. Tight glycaemic control in the DCCT attained by MDI (multiple daily injections) or CSII (continuous subcutaneous insulin infusion) was associated with a three-fold increase in SH [2].

Established risk factors for SH include age, duration of diabetes, tight glycaemic control, previous SH and impaired awareness of hypoglycaemia (IAH) [8]. IAH occurs in 20% of those with T1DM and is characterised by diminished autonomic warning symptoms of impending hypoglycaemia and associated with a six-fold increased risk of SH [9-11]. Antecedent biochemical hypoglycaemia (BH) including silent nocturnal hypoglycaemia can induce IAH in addition to diminished counter-regulatory hormone response in people with established diabetes [12].

In insulinoma patients, surgical resection restores normal symptomatic and neuroendocrine response to hypoglycaemia providing further evidence of the direct causative role of BH in IAH and SH [13]. Rigorous avoidance of hypoglycaemia by relaxing glycaemic targets while maintaining conventional MDI therapy has been shown to restore hypoglycaemia awareness with normalisation of glycaemic thresholds for symptoms and neuroendocrine responses during a stepped hyperinsulinemic–hypoglycaemic clamp study [14,15]. This was, however, associated with a 0.4–1.1% (4–12 mmol/mol) increase in HbA_1c_. Moreover, success has previously been confined to those with relatively short duration of diabetes [7] or transiently following a brief period of absolute hypoglycaemia avoidance in those with longer duration diabetes [8].

The potential for reducing nocturnal and late post-prandial hypoglycaemia by employing rapid-acting insulin analogues pre-prandially has been demonstrated [16-18]. In addition, reduced nocturnal hypoglycaemia has been reported in insulin glargine trials [19-23] and insulin detemir trials [24-27]. However, individuals with a previous history of IAH and SH and longer duration of diabetes have typically been excluded from randomized clinical trials investigating insulin analogues. The National Institute for Health and Clinical Excellence (NICE) in the UK has recommended further studies to assess the impact of insulin analogues on duration and severity of hypoglycaemia and on quality of life (QoL) [28].

In randomized trials of CSII versus MDI, a relatively modest improvement in HbA_1c_ has been demonstrated in addition to the potential for reduction in the incidence of SH [29,30]. There have been relatively few trials to date with glargine as the basal insulin in the MDI comparator group [30,31]. A Cochrane review has indicated a possible benefit of CSII as compared to MDI in reducing SH but data also indicated no benefit of CSII in reducing non-severe hypoglycaemic events [32]. Individuals with established SH have again often been excluded from prospective studies, despite a reported sustained reduction in the incidence of SH in a non-randomized, retrospective study [33]. NICE recommends CSII therapy where achievement of optimal glycaemic control has been precluded by disabling hypoglycaemia but has emphasized the absence of studies in high-risk individuals together with the need for randomized control trials to assess biomedical and psychosocial outcomes of both analogue MDI and CSII in those with established SH [34].

The potential role of real time continuous glucose monitoring (RT-CGM) has generated considerable interest among clinicians and those with T1DM since its introduction. Improved overall glycaemic control has been reported, though benefit in terms of reduced SH has not yet been demonstrated [35,36]. Studies that have compared RT-CGM integrated with CSII with analogue regimens have also failed to demonstrate a difference in rates of SH although they have suggested reduced HbA1c with the technology [37,38]. There is evidence that RT-CGM can significantly reduce the time spent with a blood glucose <3.5 mmol/l [39], however there is no evidence from the major RCTs published that it can prevent SH. This may be due to the study design and participant selection criteria of these trials. Sustained avoidance of BH achieved through feedback from RT-CGM use with the aim of restoring hypoglycaemia awareness and preventing risk of further SH in high-risk individuals with T1DM has not been assessed.

Despite implicit acknowledgement amongst healthcare professionals that SH impairs an individual’s QoL, there is little formal evidence for this in the literature. Davis *et al.,* have demonstrated the major impact of SH on perceived health and well being [40] but the full impact of SH on QoL has not been assessed adequately. Relatively few studies have directly assessed impact of successful prevention of further SH in addition to differential effects according to therapeutic intervention.

We have previously conducted a 6-month randomised prospective pilot study in individuals with T1DM complicated by SH, comparing rigorous BH avoidance with optimised analogue MDI; CSII; or education alone (EDUC) [41]. This demonstrated absolute prevention of recurrent SH in 71% in all groups. Quantitative improvement in IAH was confirmed using the validated Clarke questionnaire [42] in addition to restored symptomatic response to clamp-induced hypoglycaemia. Concomitant improvement in glycaemic control (HbA1c) was achieved with MDI (baseline: 8.6 ± 1.1%, endpoint: 7.6 ± 0.7%, p = 0.04) and CSII (baseline: 8.5 ± 1.9%, endpoint: 7.4 ± 1.0%, p = 0.06) but not EDUC (baseline: 8.5 ± 1.1%, endpoint: 8.3 ± 1.0%, p = 0.54). Significant improvements in diabetes-specific QoL and fear of hypoglycaemia were also demonstrated in MDI and CSII groups. Although in this study RT-CGM was not used, these pilot data provide the rationale, robust power calculation and proven study design for a definitive RCT, without the requirement for an education alone arm.

### Study objectives

#### Primary objective

To demonstrate that by optimising conventional management, including the use of real time continuous glucose monitoring (RT-CGM), in individuals with T1DM complicated by IAH, rigorous prevention of BH will restore awareness and reduce risk of recurrent SH.

#### Secondary objectives

To quantify and compare BH identified by self-monitored blood glucose (SMBG) and blinded CGM profiles during each intervention.

To quantify and compare overall glycaemic control and glucose lability in each group by analysis of HbA1c, SMBG and blinded CGM.

To quantify and compare total daily doses of insulin before and after the intervention period.

To compare health utility, well-being and QoL during each intervention using validated and novel measures.

To perform secondary analyses of those who continue to experience IAH regardless of study intervention, to determine factors associated with absence of response. It is hypothesised that these will include two sub-groups: one in whom an absolute focus on avoidance of high glucose (evidenced from patient-reported outcome (PRO) measures) leads to continued biochemical hypoglycaemia despite the study goals; and a second with severe autonomic neuropathy (evidenced from clinical history) who are unable to recover autonomic warning symptoms of hypoglycaemia despite effective reduction in biochemical hypoglycaemia.

To determine symptomatic, counter-regulatory hormone and cognitive response to hypoglycaemia in gold standard clamp studies. Comparisons will be made between those randomised to CSII and those randomised to MDI; in addition to RT-CGM versus no RT-CGM; and responders with restored hypoglycaemia awareness versus non-responders with persistent IAH despite study intervention.

## Methods/design

### Ethical and governance approval

Ethical approval for this study has been granted by Sunderland Research Ethics Committee (09/H0904/63) and Clinical Trial Authorisation has been given by the Medicines and Healthcare products Regulatory Agency (17136/0246/001-0001). Site Specific Approval has been granted by all participating Acute Hospital Trust Research and Development Departments.

### Study design

**Figure 1:**
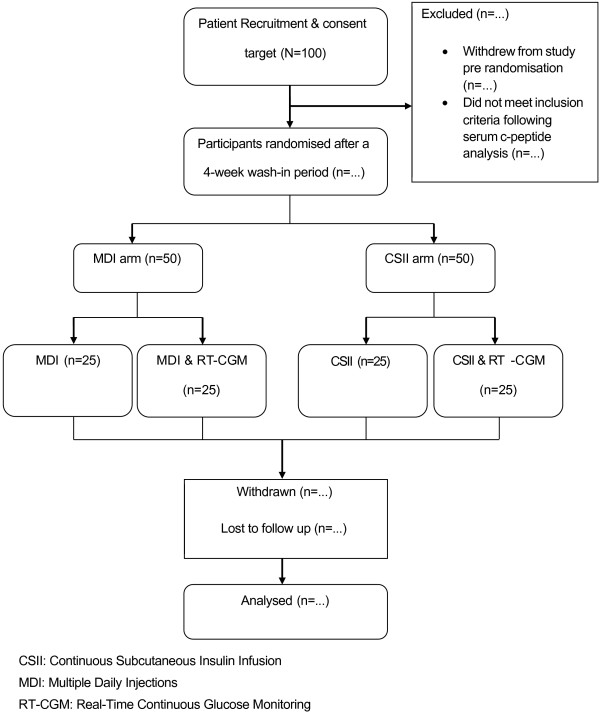
Proposed flow through RCT.

The study is an interventional multicentre prospective RCT comparing hypoglycaemia avoidance with optimised subcutaneous insulin analogue regimen (MDI) and insulin pump therapy (CSII) with or without adjunctive RT-CGM in a 2×2 factorial design (Figure 1). The trial design is consistent with the CONSORT Statement [43].

### Participants

Participants will be recruited from the diabetes clinics of the five participating UK tertiary referral and academic hypoglycaemia/CSII centres (Royal Bournemouth Hospital, Bournemouth; Addenbrooke’s Hospital, Cambridge; Newcastle Diabetes Centre, Newcastle upon Tyne, UK; Derriford Hospital, Plymouth; Northern General Hospital, Sheffield). Patients with T1DM, a history of IAH and increased risk of SH will be assessed to determine if they are eligible for the study. It is envisaged that the majority of these will have experienced SH within the preceding year, although this is not mandatory for study participation. Those who are considered potentially eligible will be approached to give their written informed consent before attending for a screening visit at which inclusion criteria will be checked.

### Inclusion criteria

Individuals who are aged 18–74 years and have a diagnosis of diabetes mellitus according to ADA [44] / WHO [45] criteria and consistent with a clinical diagnosis of T1DM. Participants will have:

serum C-peptide below the quality assured limit of detection for the assay and laboratory (<50 pmol/L) with simultaneous exclusion of BH (glucose <4.0 mmol/l) by laboratory plasma glucose assay.

impaired awareness of hypoglycaemia, as confirmed by a Gold score ≥4 [11].

### Exclusion criteria

Any condition that in the investigator’s judgement is likely to cause the participant to be unable to understand the information in the Informed Consent Document or to provide informed consent.

Insufficient proficiency in English, below that to enable the participant to understand both verbal and written information during the study. This is due to the complexity of the education programme, the need for independent completion of the questionnaire measures, and the degree of communication required between participants and clinicians during the study.

Unwilling to undertake intensive insulin therapy, including randomisation to use of CSII, optimised MDI regimen or RT-CGM.

Unwilling to undertake glucose profiles using the subcutaneous continuous glucose monitoring (CGM) equipment.

Unwilling to use SMBG at least 4 times daily.

Unwilling to monitor and record signs and symptoms of hypoglycaemia.

A history of intolerance to insulin glargine.

### Trial intervention and study procedures

Schedule for study visits is given in Table 1.

**Table 1:** Visit schedule

**Study period**	**4 week wash in period**						**24 week primary RCT**															**18 month RT-CGM RCT continuation**					
**Visit number**	**1**	**2**	**3**	**4**	**5**	**6**	**7**	**8**	**9**	**10**	**11**	**12**	**13**	**14**	**15**	**16**	**17**	**18**	**19**	**20**	**21**	**22**	**23**	**24**	**25**	**26**	**27**
informed consent	x																										
eligibility criteria	x																										
given information sheet on clamp study	x																		x								
hypoglycaemia screening questionnaire	x		x																								
HbA1c	x		x							x		x		x		x		x		x			x		x		x
C-peptide and plasma glucose	x																										
retinal photographs		x																									
urine albumin:creatinine ratio		x																									
demographic info			x																								
concomitant medication			x							x		x		x		x		x		x			x		x		x
full physical examination			x																	x							
history of glycaemic control										x		x		x		x		x									
full medical history including glycaemic control			x																	x			x		x		x
vital signs			x							x		x		x		x		x		x			x		x		x
height			x																								
weight			x							x		x		x		x		x		x			x		x		x
TFTs					x																						
Coeliac antibody					x																						
short synacthen test					x																						
detailed SH history			x							x		x		x		x		x		x			x		x		x
Modified Clarke/Edinburgh			x																	x			x		x		x
QoL questionnaires			x																	x			x		x		x
week 4 short questionnaire pack										x																	
4 week Blood Glucose / hypo diary	x		x							x		x		x		x		x		x							
7 day CGMS placement		x							x		x		x		x		x		x			x		x		x	
Autonomic function tests				x																	x						
clamp study			x																x								
education programme						x																					
Insulin administration education session							x																				
Home glucose monitoring/RT education session								x																			

### Four week baseline period

After consent, participants will undertake a 4-week wash-in period before the 24-week RCT period. Participants will be educated in the use of the study-specific prospective SMBG hypoglycaemia diary. All participants will be provided with a study hand-held glucometer (Contour link®, Bayer Healthcare) to measure daily 4-point and weekly 8-point profiles. They will be asked to record clinical details of all glucose levels <4 mmol/l and symptomatic hypoglycaemic events. All participants will wear a blinded CGM device (iPro™ 1, Medtronic) for 7 days during the baseline period.

At the end of the wash-in period, participants will attend for a baseline study visit where the investigator will complete the GCP-compliant baseline Case Report Form (CRF). This will record demographic information, full diabetes-specific clinical history including frequency and consequences of SH events over the preceding 12 months and full clinical examination assessing complication status. The study SMBG / hypoglycaemia diary will be collected and CGM device downloaded. In addition, hypoglycaemia awareness will be re-evaluated using the validated Gold Score [11] and Clarke questionnaire [42].

A first morning urine sample for albumin: creatinine ratio will be collected together with dilated retinal photography if not performed within the preceding 6 months. A blood sample will be taken for HbA1c (DCCT-aligned), urea and electrolytes, liver function tests and lipid profile analysed at local site quality-assured clinical laboratory. Study-specific PRO questionnaire booklets will be completed comprising several validated and novel measures. During design of and preparation for the study, new measures were developed by the team to capture aspects of participant experience for which validated measures did not exist (e.g. hypoglycaemia cues) or where there was specific concern that existing measures were insensitive (e.g. the Gold and the Clarke have been demonstrated to lack sensitivity to improvements in awareness). All PRO measures included in the evaluation are listed in Table 2.

**Table 2:** Validated and novel patient reported outcome measures assessed during the Hypo COMPaSS trial

**Validated PRO measures**	**Novel PRO measures undergoing validation**
• The Gold Score [44]	• The Hypoglycaemia Awareness Questionnaire (HypoA-Q)*
• The Edinburgh Hypoglycaemia Survey [54]	• The Hypoglycaemia Burden Questionnaire (HypoB-Q)* – part A only
• Clarke Hypoglycaemia Awareness Questionnaire (minimally modified version)	
• The Hypoglycaemia Fear Survey II (HFS II) [55]	• The Hypoglycaemia Cues Questionnaires (HypoC-Q)*
• The Hyperglycaemia Avoidance Scale [56]	• The Blood Glucose Monitoring Questionnaire (BGM-Q)*
• The Diabetes Treatment Satisfaction Questionnaire – status version (DTSQ(s)) [57]	• The Quality of Life Questionnaire Diabetes (QoL-Q Diabetes)^
• Insulin Treatment Satisfaction Questionnaire [58] – abridged version including two subscales only	• The Attitudes to Awareness of Hypos Questionnaire#
• EuroQoL EQ-5D [59]	
• Perceived Control of Diabetes scales (type 1) [60]	

* designed by Prof Jane Speight and Dr Shalleen Barendse (Â© AHP Research, 2010).

^ designed by Prof Jane Speight, Dr Alison Woodcock and Matthew Reaney (Â© AHP Research, 2007).

# designed by Dr Nicole DeSoyza, Helen Rogers and Prof Stephanie Amiel (King’s College London).

#### Baseline hypoglycaemic clamp study

A stepped hyperinsulinaemic hypoglycaemic clamp study will be conducted at baseline and at 24 weeks with participants who are willing to undergo this procedure for which specific consent will be sought separately. It is envisaged that approximately 25% of participants will be studied. The method is described in detail later.

#### Autonomic function testing

Before the start of the RCT, participants will attend for non-invasive detailed cardiac autonomic function testing using the recommendations in the review by Tesfaye et al. [46]. These will include heart rate response to deep breathing, a Valsalva maneuver and heart rate and blood pressure response to standing interpreted using age normative values. Spectral analysis of heart rate variability and assessment of cardiac vagal baroreflex sensitivity (BRS) will be carried out over a five minute period of controlled breathing. Structured assessment of global autonomic function will be made using the Autonomic Symptom Profile Questionnaire [47].

#### Concomitant autoimmune disease screening

Participants will attend for a short synacthen test to screen for adrenocortical insufficiency. At this visit a sample will also be taken for serum thyroid stimulating hormone assay to exclude thyroid disease and for anti-endomysial antibody analysis to exclude coeliac disease. New diagnoses of other autoimmune diseases will not preclude participation in the study. If indicated participants with newly diagnosed autoimmune disease will be referred to an appropriate specialist for further investigation and management.

#### Education visit

Following experience in the pilot study [41] and informed by insights from the qualitative study undertaken in preparation for the RCT (paper submitted), a brief education programme (with formal curriculum and workbook, referred to as the ‘My Hypo COMPaSS’ tool), was developed. Participants will attend a brief education session (approximately 3 h) individually or in small groups of up to four. During the session, a trained research fellow, specialist nurse or dietician will facilitate discussions and exercises targeted specifically at rigorous avoidance of BH while maintaining overall glycaemic control [48], including four key elements forming the four points of the ‘Hypo COMPaSS’ establishing the imperatives: to never delay the treatment of hypoglycaemia and the optimal treatments for hypoglycaemia; to recognise the individual’s unique times of increased risk; to recognise hypoglycaemia by the presence of subtle symptoms; to be particularly careful about detecting and preventing nocturnal hypoglycaemia. Also included will be advice on self-adjustment of insulin doses according to carbohydrate intake, SMBG and planned activity and recommendation for oral carbohydrate administration for all glucose levels less than 4 mmol/l.

### Intervention period

#### Randomisation

Participants will be allocated by third party concealed randomisation by centre and baseline HbA1c (with stratification cut-off of 64 mmol/mol (8%) to one of four groups for a period of 24 weeks):

MDI with SMBG (group 1; n = 25).

MDI with SMBG and RT-CGM (group 2; n = 25).

CSII with SMBG (group 3; n = 25).

CSII with SMBG and RT-CGM (group 4; n = 25).

As this is an open study it is not possible for participants to be blinded to study treatment arm. Randomisation will be administered centrally by Newcastle Clinical Trial Unit (NCTU) using a secure web based system.

#### Study Interventions

The primary goal of titration throughout the 24-week RCT period will be the absolute avoidance of all glucose levels <4 mmol/l as determined by CGM and SMBG. This will be achieved by setting ‘4 as the floor’ with all glucose levels <4 mmol/l treated by 15 g glucose with repeat SMBG every 15 min until glucose >4 mmol/l, in addition to consideration of insulin dose reduction.

The trial is designed to prevent any potential bias from additional educational support provided to those randomised to CSII or RT-CGM. All participants will be provided with a Medtronic Veo insulin pump to enable use of the bolus prandial insulin dose wizard calculator whether or not they are administering insulin by CSII. All will be provided with a Contour link SMBG meter enabling direct transmission to the pump dose calculator. Access to Carelink glucose self-management software will also be provided to all participants to be used optionally to support attainment of study targets without any specific additional goals / training.

All participants (regardless of treatment arm) will attend an education session solely on the technical aspects of the insulin administration or glucose monitoring equipment they will be using during the intervention period, i.e. participants randomised to CSII will receive education restricted to technical aspects of insulin pump management including on the need to change the infusion set at least every 72 h; participants randomised to MDI will receive education restricted to insulin device (pen) use and injection site care.

Participants randomised to RT-CGM will receive education restricted to the technical aspects of using the RT monitors including trend analysis and the use of the hypoglycaemia and hyperglycaemia alarms. They will be encouraged to wear the sensor continuously (re-siting every 7 days) but flexibly with a minimum of 7 days continuous monitoring in the last week of each month. Participants not randomised to RT-CGM will receive education restricted to the technical aspects of using the Contour link meter with the bolus calculator on the pump.

Participants will be seen one week after starting the study intervention to review progress over the first week, using glucose data to achieve the primary goal of avoiding biochemical hypoglycaemia.

### Study drugs and devices

In accordance with the Medicines for Human Use (Clinical Trials) Regulations 2004 and Directive 2001/20/EC, the drugs under investigation in this trial fall under the definition of ‘investigational medicinal product’ (IMP). Although the insulins used during this trial fall under the definition of IMPs they will be used under existing licence.

### Insulin for MDI participants

For the participants randomised to MDI, insulin aspart will be given as 3 ml cartridge 100 Units/mL in a pre-filled pen (*Flexpen®*). Insulin glargine will be given as 3 ml cartridge 100 Units/mL in a pre-filled pen (*SoloStar®*). However, for those participants who have had a previous negative experience or adverse effect with insulin aspart, insulin lispro will be offered as 3 ml cartridge 100 Units/mL in a pre-filled pen (*Kwikpen®*).

### Insulin for CSII participants

For participants randomised to CSII, insulin aspart will be the insulin used as 10 ml vial 100 Units/mL. Insulin lispro can be used instead of insulin aspart for those who have had previous negative experience / adverse effects.

### Insulin pumps

All participants will be given an insulin pump that can receive and display CGM data (Mini-Med Paradigm Veo insulin pump) and will be taught how to use the on-board bolus calculator. Only those randomised to CSII will use the pump for insulin administration, the MDI groups will only use the bolus calculator feature and, if randomised to RT-CGM, the CGM feature. All participants randomised to CSII (insulin pump therapy) will be given a single additional session restricted to technical aspects of pump management.

### Self Monitoring of Blood Glucose (SMBG)

All participants will be required to undertake daily 4-point and weekly 8-point self-monitored capillary glucose profiles. All participants will use the Contour link®, Bayer Healthcare meter and will undertake this whether or not concurrent RT-CGM is being used.

### Real time—continuous glucose monitoring

Fifty per cent of all participants will be randomised to real time monitoring, using the CE-marked REAL time continuous glucose monitor (Medtronic).

### Advice on interpretation and action from SMBG / RTCGM

All participants will attend an education session on the recording of SMBG but, for those participants randomised to RT-CGM, this session will also include the technical aspects of using the monitor. This includes trend analysis, hypoglycaemia and hyperglycaemia alarms. Participants will be given written instructions on how to use the data provided by continuous glucose monitors to make real-time adjustments of insulin doses and on the use of computer software (for those with a home computer) to retrospectively review the glucose data to alter future insulin doses. Participants randomised to RT-CGM will be encouraged to wear the sensor continuously but flexibly with a minimum of 7 days continuous monitoring in the final week of each month. Those participants randomised to RT-CGM will be advised to re-site the sensor every 7 days.

### Blinded continuous glucose monitors

Blinded CGM will be undertaken using the CE-marked Medtronic wireless iPro system. These will be used in the 4-week wash in period and during the last seven days of every month. The patients and investigators are blinded to all of this data until the end of the primary 24 week RCT.

### Insulin titration protocol

The blood glucose targets (for all patients in CSII and MDI groups) will be as follows:

Fasting blood glucose (FBG): 5.0 - 7.0 mmol/l.

Pre-prandial blood glucose: 4.5 - 7.0 mmol/l.

Post-prandial glucose*: 6.0 – 8.0 mmol/l.

Bedtime blood glucose**: 6.0 – 8.0 mmol/l.

4 am blood glucose: 5.0 – 7.0 mmol/l.

*postprandial blood glucose: measurement made 2 h after the start of a meal.

**bedtime blood glucose: measurement made within 30 min of retiring to bed for the night.

### Glargine titration in MDI group

Insulin glargine will be self-administered and the following titration protocol will be followed:

Take within 30 min of retiring to bed for night / no need for snack.

Aim for stable (not falling) glucose through the night.

Reduce dose if any hypoglycaemic episodes or glucose <5.0 mmol/l between 4 am and before breakfast.

Target glucose of 5–7 mmol/l before breakfast—adjust dose by 1–2 units to maintain target if necessary with primary aim being absolute avoidance of BH (Biochemical Hypoglycaemia).

During periods of illness, basal insulin doses may need to be altered and this will be guided by SMBG levels.

### Introduction of twice daily glargine

Participants randomised to MDI already on twice daily glargine will continue on this from the outset of the RCT. In other MDI participants, if glucose is consistently >7 mmol/l before evening meal or highly variable between breakfast and evening meal, add second dose of insulin glargine before breakfast. Initial dose will be 4 units but can be adjusted in light of participant’s current insulin doses. If glucose has been falling through the night, a 2–4 unit reduction in evening glargine dose will be actioned before bed on the day of commencing the morning dose. The addition of a second daily glargine dose will be considered for all participants in the MDI group. This can be initiated between study visits if necessary, e.g. after telephone advice.

Morning insulin glargine will be self-administered and adjusted as follows:

Take within 30 min of rising from bed for the morning.

Aim for stable (not falling) glucose through the afternoon.

Reduce dose if any hypoglycaemic episodes or glucose <5 mmol/l between 2 h after lunch and evening meal.

Target glucose of 5-7 mmol/l before evening meal – adjust dose by 1–2 units to maintain target if necessary with primary aim being absolute avoidance of BH.

### Basal insulin titration in CSII group

The basal insulin delivery rate will be titrated according to fasting, bedtime, pre-prandial and 4 am glucose levels ensuring absence of recurrent low glucose levels at these times (checkpoints). Increased or decreased delivery will be commenced from the previous basal insulin checkpoint level, i.e. if low at 4 am—decrease from bedtime; if high fasting increase from 4 am.

Mean fasting; bedtime; 4 am and pre-prandial blood glucose:

**Within target**: No change to basal delivery rate.

**Above target**: Increase basal insulin by 0.1 U/hr from previous check point.

**Below target or unexplained late post-prandial hypoglycaemia**: Decrease basal insulin by 0.1 U/hr from previous check point.

During periods of illness, basal insulin rates may need to be altered and this will be guided by SMBG levels.

### Meal-time insulin bolus in all groups (CSII and MDI)

Carbohydrate counting skills and bolus dose adjustment in light of current blood glucose level / individualised insulin carbohydrate ratios will be reviewed in all participants. Aspart or lispro will be delivered either by subcutaneous injection or as a subcutaneous pump bolus before all meals and snacks with substantial carbohydrate content.

Insulin: carbohydrate ratios will be calculated for all individuals using the ‘500 rule’ and using total daily insulin doses pre-randomisation. The ‘500 rule’ is:

500 divided by the TDD (Total Daily Dose of insulin) = grams of carbohydrate covered by one unit of aspart or lispro.

In the event of high pre-prandial glucose levels corrective doses will also be recommended with meals as part of the meal time bolus. This will be calculated using the ‘100 rule’ for estimation of Insulin Sensitivity Factor. The ‘100 rule’ is:

100 divided by the TDD (Total daily Dose of insulin) = glucose drop in mmol/l per 1 unit of aspart or lispro.

This will be presented to all participants as ‘*1 unit of aspart / lispro will reduce your blood glucose by x mmol/l*’.

Corrective doses with all pre-main meal boluses / prandial insulin injections will be encouraged according to the 100 rule when glucose level is above target.

The insulin: carbohydrate ratio and Insulin Sensitivity Factor for that period of the day will be adjusted accordingly in the event that:

The glucose level is consistently below or above target 2 h after a bolus / prandial insulin injection.

If any unexplained hypoglycaemic event occurs 2 h after a bolus / prandial insulin injection.

### Telephone contact

Participants will be contacted by telephone daily for the first week after starting the study intervention and thereafter weekly throughout the RCT to reinforce the primary goal of BH avoidance, provide clinical review / support, and ensure diary completion.

### Study follow up

Participants will attend for a study visit every four weeks during the RCT for collection of SMBG/hypoglycaemia diary and HbA1c. One week prior to each visit participants will have a blinded CGM device fitted and these data will be downloaded at the visit. Participants will have their weight measured at each follow up visit and this, along with details of insulin dosage, will be recorded on visit specific CRFs. Both investigator and participant will remain blinded to the results of the blinded CGM data during the RCT period. Clinical review at each follow up visit will reinforce the primary goal of BH avoidance. At each follow up visit information will be collected on any episodes of hypoglycaemia experienced, duration of RT-CGM monitoring usage and RT-CGM alarm settings.

At week 24, participants will attend for the primary RCT completion visit. This will include blinded CGMS data download, collection of SMBG/hypoglycaemia diary, and HbA1c. Participants will also be asked to complete the ‘end of RCT’ study-specific questionnaire booklets assessing hypoglycaemia experience and other PROs (Table 2).

On the same day, participants will be invited to attend for ‘end of RCT’ stepped hyperinsulinaemic hypoglycaemic clamp as described below.

At the end of this intervention period, participants will be asked to attend for repeat detailed cardiac autonomic function testing and subjective assessment of global autonomic symptoms.

### Post primary RCT follow-up

At the end of the 24-week RCT, participants will return to routine clinical care. Those randomised to CSII may stop this if they and their clinical team wish. Commencement of CSII according to NICE guidance [34] will be considered in those previously randomised to MDI. Those participants who were randomised to RT-CGM will continue with this intervention for a further 18 months constituting an overall 24 month RCT of RT-CGM augmented glucose monitoring vs SMBG alone. RT-CGM will not be offered to those not randomised to this intervention, as it is not currently recommended by NICE. All participants will be invited to attend three follow-up visits 6, 12 and 18 months post primary RCT (Table 1). Participants will be fitted with a blinded CGM device for 7 days before each follow-up visit. This visit will include collection of SMBG/hypoglycaemia diary; SMBG / CGM data download, HbA1c measurement. Participants will be required to complete questionnaire booklets (Table 2) at each follow-up visit.

The end of the study will be the last follow-up visit of the last participant scheduled for 24 months after the commencement of study interventions.

### Hypoglycaemic clamp study

A stepped hyperinsulinaemic hypoglycaemic clamp study [49] will be conducted in participants who are willing to undergo this procedure for which separate informed consent will be obtained. It is envisaged that approximately 25% of participants will be studied. Additional exclusion criteria will be in place to ensure participant safety:

Age >60 years.

History of epilepsy (seizures not primarily induced by hypoglycaemia).

Known ischaemic heart disease.

Other significant disease which in the judgement of the investigator precludes participation.

Participants will be fitted with a retrospective CGM sensor to be worn typically for five to seven days (at least 24 h) preceding the study day. This will be downloaded on the morning of the study to determine whether any antecedent biochemical hypoglycaemia (BH) occurred over the 24 h period prior to the clamp. Studies will be postponed to another day if any CGM and/or self-monitored capillary glucose below 3.0 mmol/l are detected during the preceding 24 h. For participants who required rescheduling, a further 72 h of CGM will be organised. All participants will be advised to fast from 22:00 h and to avoid caffeine for 24 h before the study.

The participant will be admitted to the clinical research facility at 7 am on the day of the study. On arrival, an intravenous cannula will be inserted in the ante-cubital vein of the non-dominant arm and blood glucose will be stabilized using sliding scale insulin infusion aiming initially for blood glucose 6.0–7.0 mmol/l and then 5.0–6.0 mmol/l between 10.30 am and 11 am for clamp initiation.

A second retrograde cannula will be inserted into a vein on the dorsum of the non-dominant hand for the sampling of arterialised venous blood. The hand will be kept in a purpose-built heated box (Temp 50–60°Celsius) before inserting the retrograde cannula and throughout the clamp study. A slow intravenous infusion of saline will be used as needed to keep the sampling line patent. During this period of stabilization, participants will be shown how to perform specific cognitive function tests (Four choice reaction time [50-52] and Stroop tests [53,54]) and asked to practise the tests until they achieve consistent results (typically 5 practice sessions). Four-choice reaction time is a test of attention, discrimination and motor speed reaction while Stroop tests are a group of related sub-tests all requiring selective attention and mental tracking.

At the start of the clamp a primed infusion of 60 mU/m^2^/min soluble human Actrapid insulin will be started via the non-dominant antecubital vein catheter. Dextrose infusion rates will be adjusted as needed, aiming to stabilise plasma glucose at 5.0 mmol/l at 40mins followed by step-wise lowering to 3.8 mmol/l, 3.4 mmol/l 2.8 mmol/l and 2.4 mmol/l. Each step will last 40 min allowing 20 min to achieve new target and 20 min for stabilization at that level. Samples for plasma glucose will be obtained every 5 min and analysed in real-time but participants will be blinded to these glucose levels throughout the study.

At the end of each clamp stage participants will be asked to complete a validated symptom questionnaire [55,56] followed by the four choice reaction time test and Stroop test. Each symptom will be graded on a visual analogue scale from 1 (not at all) to 7 (very severe).

Arterialised venous blood samples for insulin, catecholamines, growth hormones, glucagon and cortisol will be obtained every 10 min during the first 40 min of the study (euglycaemia) followed by every 20 min during progressive hypoglycaemia. Heart rate and blood pressure will be recorded every 20 min. In addition, spectral analysis of heart rate variability and assessment of cardiac vagal baroreflex sensitivity (BRS) will be carried out during each clamp stage. At the end of the study, insulin infusion will be reduced to basal insulin requirements and dextrose infusion increased to raise blood glucose to euglycaemia. Participants will be provided with lunch with post-meal insulin bolus and re-established on their usual insulin regimen thereafter.

### Sample size

The recruitment target for the overall RCT is n = 100 participants (n = 20 from each of the five participating centres). From the pilot study data [41], the sample size of n = 100 (n = 25 in each of the four study arms) would give 80% power at a significance level of 0.05 to detect a difference of 1.1 between the IAH scores (assessed using the Gold Score) of the 50 participants randomised to either of the CSII arms and the 50 randomised to either of the MDI arms. A difference of at least one point (on the 7-point scale) is considered to be a clinically relevant change on the Gold Score. The calculation is based on the use of the 2-sample t-test and the assumption, taken from the pilot data, that the standard deviation of the IAH score is 2 (mean ± SD Gold Score in the pilot study was 2.57 ± 1.90 in the CSII arm and 4.0 ± 1.79 in the MDI arm).

### Outcomes / statistical analysis

The principal analysis will examine the factorial structure of the treatment and monitoring regimen effects on the difference in IAH (Gold score) at 24 weeks using analysis of covariance (ANCOVA). Baseline IAH (Gold score) and stratification (centre and baseline HbA1c) variables will be included among the covariates to be considered in addition to suitable summaries of questionnaire scores and glucose monitoring data collected at baseline prior to randomisation. The glucose monitoring data to be collected include time spent for the following separate ranges: <2.5 mmol/l, <3 mmol/l, <4 mmol/l, >7 mmol/l, >10 mmol/l, between 4 and 7 mmol/l and between 3 and 10 mmol/l. The inclusion of baseline HbA1c as a covariate will enable the examination of possible interactions between effects observed and these values.

Further analyses will be undertaken concerning IAH to corroborate the Gold Score; the Gold Score will be compared with scale and subscale scores derived from the Clarke Questionnaire and the Hypoglycaemia Awareness Questionnaire (HypoA-Q) at 24 weeks.

These measures will also be subject to analysis as for the primary outcome. Additionally, a binary indicator of IAH response (defined as a Gold Score of <4 or ≥4) at 24 weeks will be analysed using logistic regression making use of the covariates used for the primary outcome analysis.

There will also be an additional analysis of the (paired) change in IAH (Gold Score) over the 24-week duration of the trial using the t-test without consideration of the intervention or monitoring groups in order to evaluate the effect of undergoing any intervention or monitoring over the 24-week period.

Other outcomes will be assessed at baseline and 24 weeks. Analysis methods will generally be similar to that described for the primary analysis but alternative techniques such as McNemar’s test and logistic regression will be used as appropriate.

Further analyses will be undertaken using HbA1c and the separate continuous glucose monitoring measures (time spent in the following separate ranges: <2.5 mmol/l, <3 mmol/l, <4 mmol/l, >7 mmol/l, >10 mmol/l, between 4 and 7 mmol/l and between 3 and 10 mmol/l) as outcome variables.

Similar analyses will be undertaken on scores from all PRO measure scores used in the study (Table 2).

A number of measures relating to SH (ADA criteria) will be analysed: number of episodes of SH at 24 weeks, change in SH between baseline and 24 weeks (reported as difference in annualised rate pre and post-intervention), change in SH between baseline and 24 weeks (reported as the proportion of participants with reduction in number of SH events compared between the timepoints) and change in proportion without SH between baseline and 24 weeks.

Changes in weight, total daily dose of insulin, and in glucose lability will be subject to analysis in a similar manner to the primary outcome.

Wherever possible participants who elect to withdraw from the study will be followed up so that final outcome data are obtained, enabling their inclusion in an Intention to Treat (ITT) analyses. This will form the analysis groups for the analyses described above.

Analyses restricted to those participants who were allocated to use RT-CGM will be considered, in order to allow use of the further covariate of low or high CGM use (defined by consideration of a pre-defined cut-off value) throughout the 24-week period. Variables analysed in this manner will include IAH (Gold Score), episodes of SH, HbA1c and several of the glucose monitoring measures.

Variables with missing data will be examined and the amount of missing data described. Data analysis will take the form of a complete case analysis, although imputation of values may be considered for the primary outcome variable alone should this be missing to a sufficient extent.

Significance levels will be set at α=0.05 throughout.

Safety data are to be documented but will not be subject to statistical analysis.

A detailed analysis plan will be finalised prior to the commencement of data analysis.

Data from the clamp study will be analysed separately; however a comparison will be made between the IAH (Gold) based definition of response and a response measure derived from the clamp results.

### Trial Governance

Trial management will be overseen by a Trial Management Group who will meet regularly to discuss the operational aspects of the trial. An independent Data Monitoring and Ethics Committee (DMEC) will be convened to undertake independent review, monitoring safety and efficacy endpoints. The DMEC will comprise two physicians not connected to the trial (at least one of whom will have expertise in hypoglycaemia), one statistician and one patient representative. The DMEC will have full access to unblinded study data.

A Trial Steering Committee (TSC) will supervise the trial, ensuring it is conducted to high standards in accordance with the protocol, the principles of GCP, and with regard to participant safety. This committee will have an independent chair with expertise in hypoglycaemia. In addition to the Chief Investigator (Professor James Shaw) and Principal Investigators, the TSC will consist of a sponsor/funder representative, representatives of the Newcastle Clinical Trials Unit and two consumer representatives. The TSC will also consider safety issues for the trial and relevant information from other sources, ensuring at all times that ethical considerations are met when recommending the continuation of the trial.

## Discussion

Over recent years, it has become increasingly clear that SH is the major factor limiting the overall level of glucose control achievable in those with established T1DM. We believe that this study will provide definitive evidence that recurrent SH is preventable even in the majority of those at highest risk. This will enable a truly evidence-based approach to improving day-to-day diabetes management for all those currently living with the risk and fear of SH. In addition, characteristics of the minority in whom optimised conventional therapy is likely to prove inadequate will be defined, enabling early identification and intervention.

While the interventions in this trial are widely used in an attempt to reduce the incidence of SH, there is little evidence from adequately powered RCTs supporting their use, with most existing RCTs having been powered for change in glycated haemoglobin rather than SH or IAH. The primary objective of this trial is to determine whether these treatments improve awareness of hypoglycaemia and, secondarily, to determine whether they have an impact on SH events and overall glycaemic control. Crucially, this unique trial will also determine the impact of these intensive interventions on a range of patient-reported outcomes.

The trial does pose challenges, perhaps foremost the need to ensure equal input to all study participants and not provide additional care to those randomised to pump therapy. The protocol was designed to take this into consideration. Participants randomised to MDI will also be provided with insulin pumps so that this group is not disadvantaged by not having access to a bolus calculator. Secondly, study investigators will need to remain absolutely focused on biochemical hypoglycaemia avoidance, undiluted by attempts to tighten or maintain overall glycaemic control. This may be made more challenging by existing investigator and participant behaviours. For this reason, detailed insulin titration protocols (detailed above) will be used across all sites.

This ambitious and intensive trial will demonstrate definitively whether SH, a cause of major morbidity in up to 5000 adults with T1DM in the UK, can be prevented successfully in even those at highest risk by optimised conventional management using existing technology. Overall biomedical, psychosocial and health utility impact of MDI, CSII and RT in this group will be determined employing optimised measures as detailed above.

## Competing interests

No pharmaceutical company or medical device manufacturer has had any role in the design or funding of this trial.

DK has received honoraria for participation in educational events and consultancy fees from Abbott Diabetes Care, manufacturers of glucose sensors.

JAMS has in the past taken part in Medical Advisory Boards for Novo Nordisk, Sanofi Aventis, Johnson and Johnson and Medtronic.

JSp is a member of the Accu-Chek Advisory Board. In relation to this activity, JSp’s research group, The Australian Centre for Behavioural Research in Diabetes, has received consultancy fees from Roche Diagnostics Australia. It is also the recipient of unrestricted educational grants from Sanofi Aventis and Medtronic, and speaker fees from Abbott Diabetes Care.

PC has received speaker fees and been on Advisory Boards for Medtronic, Johnson and Johnson and Roche.

ME has acted on Advisory Boards for Medtronic, Roche and Cellnovo and received travel support/speakers fees from Medtronic and Animas.

SH has carried out consultancy work for pump/meter, insulin companies, Lifescan, Sanofi-Aventis, NovoNordisk and Lilly. Medtronic has supplied pumps for use in one of his studies.

## Authors’ contribution

JAMS, JSp, DK, PC, SH, ME, DF, TO, RT, SMM and SB conceived the study and participated in its design. TC advised on the statistical analysis plan. SL, LL, HKT and EW have responsibility regarding the clinical management of the participants and helped to draft the manuscript. CB and JSt participated in the study design and coordination. All authors read and approved the final manuscript.

## Pre-publication history

The pre-publication history for this paper can be accessed here:

http://www.biomedcentral.com/1472-6823/12/33/prepub
